# Splitting of the Alveolar Process of the Lower Jaw

**Published:** 1858-12

**Authors:** G. J. Fisher

**Affiliations:** Sing Sing, N.Y.


### SPLITTING OF THE ALVEOLAR PROCESS OF THE LOWER JAW.

PEEKSKILL, N. Y., Nov. 1, 1858.

MESSRS. EDITORS:—I send you the following communication, which I received from G. J. Fisher, M. D., of Sing Sing, New York, eminent as a surgeon in this part of the country, which you are at liberty to publish, if you think it of sufficient interest to the dental profession, to be intitled to a place in your valuable journal. Respectfully, yours,

E. D. FULLER, D. D. S.

SING SING, N. Y., October 28, 1858.

MY DEAR FRIEND:—I send you an account of a case which is so near the boundary line which separates dental from general surgery, that you may be interested with it, and even consider it worthy of insertion in some one of the journals devoted to your speciality. You are at liberty to use it as your own.

Miss V., of Tarrytown, New York, a tall and comely lass, while engaged in juvenile sports with several children in the dining room, was tripped by her dress, causing her to fall, with great violence, striking the front teeth of the lower jaw against the margin of a mahogony table. On arising, she found the teeth driven into the mouth, and the jaw broken.

She applied to a physician, who directed her to a dentist for treatment. She came to Sing Sing the following day (Oct. 20th), and applied to Dentist Greenleaf, who, after examining the case, came to the conclusion that it was a little more surgical than dental, and therefore called me in to treat the injury.

Upon examination, I found the whole of the incisors and left cuspidati of the lower jaw had been driven into the mouth, splitting the alveolar process as low as the extremity of the fangs; the right lateral incisor had been detached and removed,—the remaining incisor teeth, and the eye tooth, were held together by the mass of alveolar process, which had been split off; the whole, teeth and bone, moved together, forward aud backward in the mouth; they were depressed one-sixth of an inch below their natural position.

A band of silver plate, two lines wide, and long enough to reach to the bicuspids on either side of the jaw, was bent in the form of the lower dental arch. This band was perforated with small holes, opposite the spaces between each tooth. A fine-drawn silver wire was used for loops to surround the necks of the bicuspids, passing through the perforations in the band, which was placed in front of the teeth, and the ends of the loops twisted tightly. The mass of alveolar, and the teeth which were attached to it, were elevated and placed in *situ naturale*, and retained by loops of silver wire passed around the neck of each tooth, and through the perforations in the band, and tightly twisted in front. When completed, the mouth presented its usual contour, and the dental arch was regular and firm.

I have since seen the case, which was progressing favorably, with every prospect of perfect union.

I am not much posted in dental lore, and may be thought childish for reporting so simple a case, and the treatment of which is so plain and simple, and so likely to be suggested at a glance. Yours, truly, G. J. Fisher, M. D.

P. S. The mahogony table was but slightly injured, although it was deeply impressed with the force of the young lady's character, as it now exhibits the distinct imprints of four of her teeth. G. J. F.

